# The Effects of High Molecular Weight Hyaluronic Acid Eye Drop Application in Environmental Dry Eye Stress Model Mice

**DOI:** 10.3390/ijms21103516

**Published:** 2020-05-15

**Authors:** Takashi Kojima, Taeko Nagata, Haruka Kudo, Wolfgang G. K. Müller-Lierheim, Gysbert-Botho van Setten, Murat Dogru, Kazuo Tsubota

**Affiliations:** 1 Department of Ophthalmology, Keio University School of Medicine, Tokyo 160-8582, Japan; kojima_takashi@keio.jp (T.K.); tngtax4@gmail.com (T.N.); kudo_haruka@chukyogroup.jp (H.K.); tsubota@z3.keio.jp (K.T.); 2 i.com medical GmbH, 81241 Munich, Germany; ml@coronis.net; 3 Karolinska Institutet, Dept of Clin NeuroSci, St Eriks Eye Hospital, 11282 Stockholm, Sweden; gysbert-botho.vansetten@ki.se

**Keywords:** dry eye, high molecular weight hyaluronic acid, treatment, corneal subbasal dendritic cell density, Muc5AC, ocular surface staining

## Abstract

Hyaluronic acid (HA) ophthalmic solution is widely used in dry eye treatment worldwide. However, there are no reports comparing the dry eye treatment effects of high molecular weight HA with low molecular weight HA. Sixty eight-week-old C57BL/6 mice were assigned to the following 6 groups and exposed to environmental dry eye stress (EDES) that mimics office work environment: (1) 0.1% low molecular weight HA (LMWHA) eye drops, (2) 0.3% LMWHA eye drops, (3) 3% diquafosol sodium (DQ) eye drops, (4) 0.15% high molecular weight HA (HMWHA) eye drops, (5) no treatment with exposure to EDES (EDES+/Treatment−), and (6) no treatment without exposure to EDES (EDES−/Treatment−). After EDES, the HMWHA group had significantly longer break-up time (BUT) than the 0.1%, 0.3% LMWHA groups and the DQ group. After EDES, the HMWHA group had significantly lower lissamine green staining scores than the LMWHA and DQ groups. Subepithelial presumed dendritic cell density in the HMWHA group was significantly lower than the EDES+/Treatment− group. After EDES exposure, Conjunctival Muc5AC mRNA expression in the HMWHA group was significantly higher than the 0.1 and 0.3% LMWHA groups. Ophthalmic HMWHA solution may have a better dry eye treatment effect than LMWHA or DQ solution, owing to its anti-inflammatory effect.

## 1. Introduction

Large epidemiological studies have reported a dry eye prevalence of 10.1% for men and 20.1% for women in office workers [1]. It is known that dry eye in office workers is accompanied by eyestrain and visual function deterioration, which ultimately affects work productivity [2]. Therefore, treatment and prevention of dry eye seems to be an important health problem for office workers. It has also been reported that dry eyes can be worsened by wearing contact lenses in office workers. It has been pointed out that dry eye in visual display terminal (VDT) workers is associated with lacrimal gland dysfunction [3] or a decrease in MUC5AC concentration in tears [4]. This is in accordance with a previous study which reported desiccating stress induced T cell-mediated inflammation of the cornea, conjunctiva, and lacrimal gland, including decreased tear secretion and loss of conjunctival goblet cells in mice [5,6].

Tear film oriented therapy has been recommended as a treatment strategy for dry eye by the Japanese Dry Eye and Asia Dry Eye Societies [7,8,9,10]. The concept is examining where the abnormality in the tear film is and performing a stratified treatment based on the examination findings. Treatments for the aqueous layer of the tear film include artificial tears, secretagogues, and punctal plugs. Artificial tears have long been used in the treatment of dry eye and have the effect of improving the aqueous layer by increasing the retention of water. In Japan, sodium hyaluronate ophthalmic solutions have for many years been used to treat dry eye.

We have previously reported an environmental dry eye stress (EDES) model as an animal model that mimics the visual display terminal work [11]. EDES is a mouse model of the modern office worker environment, inducing dry eyes due to spatial and air conditioning stress. Animals when exposed to EDES develop inflammation mediated loss of tear volume and secretory Muc5AC, instable tear film resulting in decrease of tear break-up time (TBUT), and ocular surface damage.

Hyaluronic acid (HA) is a member of the family of glycosaminoglycans that is a major component of the extracellular matrix, and plays an important role in organizing and maintaining tissues. It has also been reported to be deeply involved in each stage of wound healing [12]. Therapeutic effect of HA has previously been proven [13], and it has been widely used as a treatment for dry eyes. In animal experiments, it has been reported that the effect on promoting wound healing in corneal erosions is higher than that of vehicles [14]. Both low and high molecular weight HA eye drops are increasingly being used in ophthalmology. It has been reported that the high molecular weight HA (HMWHA) has anti-inflammatory and wound healing-promoting effects as compared to low molecular weight HA (LMWHA) [15,16,17,18]. A previous clinical study has also shown that high-molecular-weight HA eye drops can be used as an alternative to autologous serum eye drops for severe dry eyes [19].

The purpose of this study was to compare 0.15% HMWHA eye drops with 0.1 and 0.3% LMWHA eye drops, as well as 3% diquafosol sodium (DQ) with respect to their effectiveness to prevent and treat EDES induced dry eye disease in mice.

## 2. Results

### 2.1. Aqueous Tear Secretion and Tear Film Stability

The amount of tear secretion and tear film stability were measured 3 days before EDES exposure, on the day before EDES exposure, after 3 days of EDES exposure, and 4 days after termination of EDES exposure.

**Figure 1:**
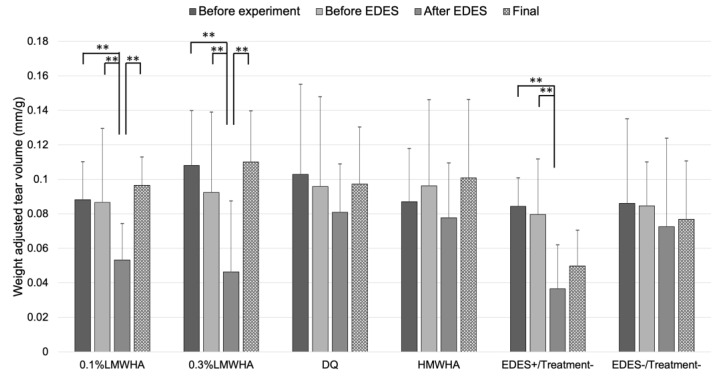
Time-course changes of weight-adjusted tear volume. In the 0.1% LMWHA, 0.3% LMWHA, and EDES+/Treatment− groups, tear volume significantly decreased after exposure to EDES. In contrast, in the DQ, HMWHA, and EDES−/Treatment− groups, there were no significant changes after exposure to EDES. ** represents *p* < 0.001. LMWHA, low molecular weight hyaluronic acid (HA); DQ, diquafosol sodium; HMWHA, high molecular weight HA; EDES, environmental dry eye stress.

Tear secretion volume significantly decreased in the 0.1% LMWHA, 0.3% LMWHA and EDES+/Treatment− groups. In contrast, tear secretion volume in the DQ and HMWHA groups did not show significant changes (Figure 1).

**Figure 2:**
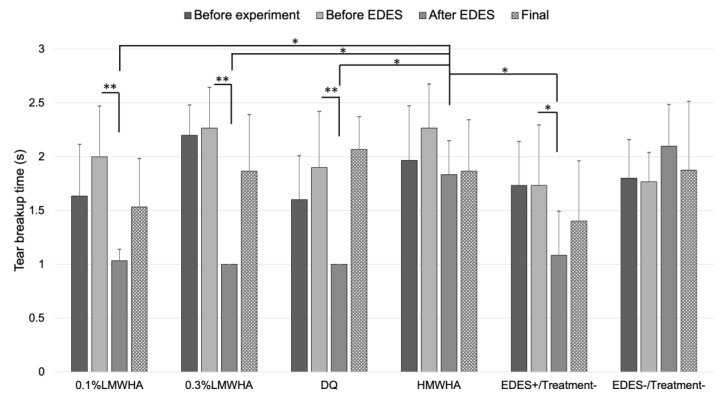
Time-course changes of tear breakup time (BUT). In the 0.1% LMWHA, 0.3% LMWHA, DQ, and EDES+/Treatment− groups, tear BUT significantly decreased after exposure to EDES. In contrast, in the HMWHA and EDES−/Treatment− groups, there were no significant changes after exposure to EDES. Tear BUT in the HMWHA after exposure to EDES was significantly longer than 0.1% LMWHA, 0.3% LMWHA, DQ, and EDES+/Treatment− groups. * and ** represent *p* < 0.05 and *p* < 0.001, respectively. LMWHA, low molecular weight hyaluronic acid (HA); DQ, diquafosol sodium; HMWHA, high molecular weight HA; EDES, environmental dry eye stress.

In the 0.1% LMWHA, 0.3% LMWHA, DQ, and EDES+/Treatment− groups, TBUT significantly decreased after exposure to EDES (0.1% LMWHA group, 0.3% LMWHA group, DQ group: *p* < 0.001, EDES+/Treatment− group: *p* = 0.012) (Figure 2). TBUT in the HMWHA group after EDES exposure was significantly longer than in the 0.1% LMWHA, 0.3% LMWHA, DQ, EDES+/Treatment− groups (vs. 0.1% LMWHA, *p* = 0.033; vs. 0.3% LMWHA, *p* = 0.042; vs. DQ, *p* = 0.044; vs. EDES+/Treatment−, *p* = 0.028).

### 2.2. Changes in Vital Staining Score

The vital staining scores, including the fluorescein and the lissamine green staining scores, were evaluated 3 days before EDES exposure, on the day before EDES exposure, after 3 days of EDES exposure, and 4 days after termination of EDES exposure.

**Figure 3:**
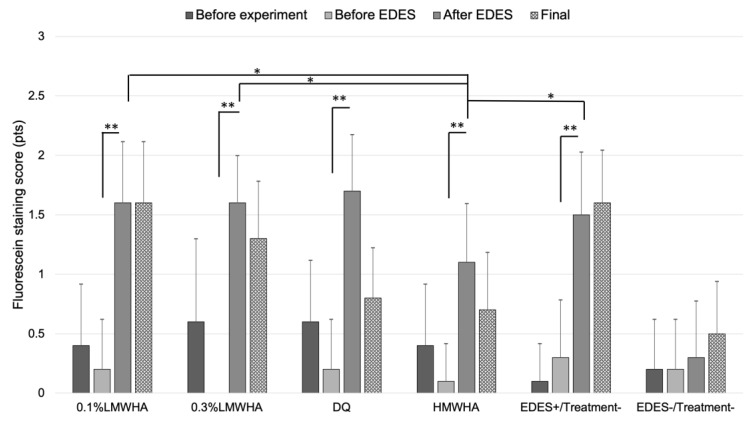
Time-course changes of fluorescein staining score. In the 0.1% LMWHA, 0.3% LMWHA, DQ, HMWHA, and EDES+/Treatment− groups, the mean fluorescein staining score significantly increased after exposure to EDES. The mean fluorescein staining score in the HMWHA after exposure to EDES was significantly lower than 0.1%LMWHA, 0.3% LMWHA, and EDES+/Treatment− groups. * and ** represent *p* < 0.05 and *p* < 0.001, respectively. LMWHA, low molecular weight hyaluronic acid (HA); DQ, diquafosol sodium; HMWHA, high molecular weight HA; EDES, environmental dry eye stress.

The mean fluorescein staining score significantly increased after exposure to EDES in the 0.1% LMWHA, 0.3% LMWHA, DQ, HMWHA, and EDES+/Treatment− groups (Figure 3). The mean fluorescein staining score of the HMWHA group after EDES was significantly lower than that in the 0.1% LMWHA (*p* = 0.025), the 0.3% LMWHA (*p* = 0.034), and the EDES+/Treatment− groups (*p* = 0.016).

**Figure 4:**
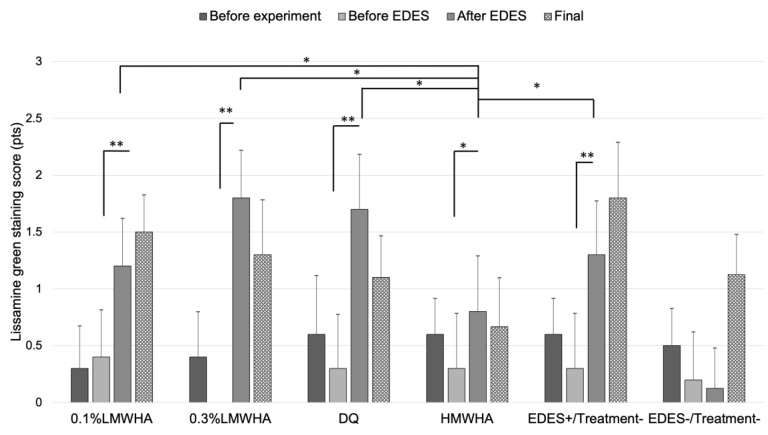
Time-course changes of lissamine green staining score. In the 0.1% LMWHA, 0.3% LMWHA, DQ, HMWHA, and EDES+/Treatment− groups, the mean lissamine green staining score significantly increased after exposure to EDES. In contrast, in the EDES−/Treatment− group, there were no significant changes after exposure to EDES. The mean lissamine green score in the HMWHA was significantly lower than 0.1% LMWHA, 0.3% LMWHA, DQ, and EDES+/Treatment− groups after exposure to EDES. * and ** represent *p* < 0.05 and *p* < 0.001, respectively. LMWHA, low molecular weight hyaluronic acid (HA); DQ, diquafosol sodium; HMWHA, high molecular weight HA; EDES, environmental dry eye stress.

The mean lissamine green staining score was significantly worse after EDES in the 0.1% LMWHA, 0.3% LMWHA, DQ, HMWHA, and EDES+/Treatment− groups (Figure 4). The mean lissamine green staining score in the HMWHA group after EDES exposure was significantly lower than the 0.1% LMWHA (*p* = 0.044), 0.3% LMWHA (*p* = 0.012), DQ (*p* = 0.028), and EDES+/Treatment− groups (*p* = 0.013).

### 2.3. In Vivo Confocal Microscopy Evaluations

**Figure 5:**
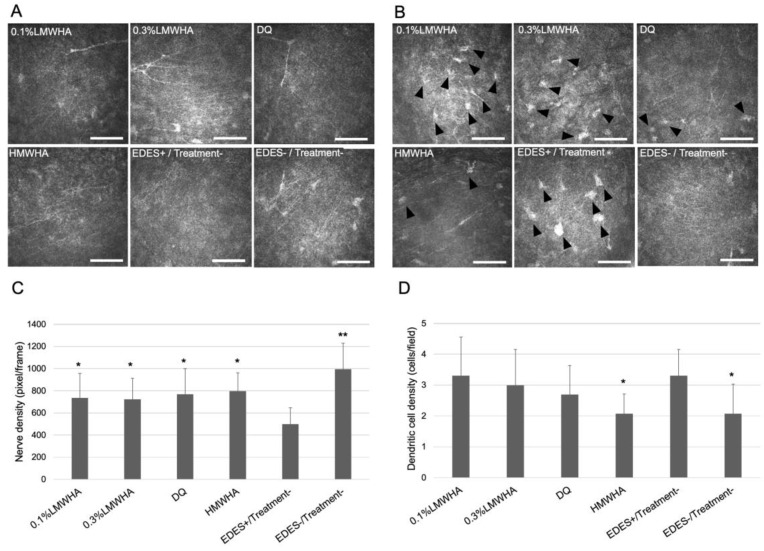
Comparison of subepithelial nerve density and presumed dendritic cell density measured by in vivo confocal microscopy. (**A**) Representative confocal microscopy image showed marked decrease of subepithelial nerve in the EDES+/Treatment− group. (**B**) Representative confocal microscopy image showed marked increase of subepithelial dendritic cell infiltration in the EDES+/Treatment− group. (**C**) The mean subepithelial nerve density in the EDES+/Treatment− group was significantly lower than other groups. (**D**) The mean subepithelial dendritic cell density in the HMWHA and EDES−/Treatment− groups was significantly lower than the EDES+/Treatment− group. * and ** represent *p* < 0.05 and *p* < 0.001 when each group was compared with the EDES+/Treatment− group, respectively. Scale bar shows 100 µm. Black arrowheads show presumed dendritic cells. LMWHA, low molecular weight hyaluronic acid (HA); DQ, diquafosol sodium; HMWHA, high molecular weight HA; EDES, environmental dry eye stress.

Subbasal nerve density and presumed dendritic cell density were evaluated using the in vivo confocal microscopy on the fourth day after EDES exposure was completed (Figure 5). In the 0.1% LMWHA group (*p* = 0.023), 0.3% LMWHA group (*p* = 0.028), DQ group (*p* = 0.032), HMWHA group (*p* = 0.046), and EDES+/Treatment− group (*p* < 0.001), the mean subbasal nerve density was significantly higher than in the EDES+/Treatment− group. The mean dendritic cell density in the HMWHA and EDES−/Treatment− groups was significantly lower (*p* = 0.033) than the EDES+/Treatment− group (vs. HMWHA, *p* = 0.033; vs. EDES−/Treatment−, *p* = 0.027).

### 2.4. Conjunctival Muc5AC Alterations

**Figure 6:**
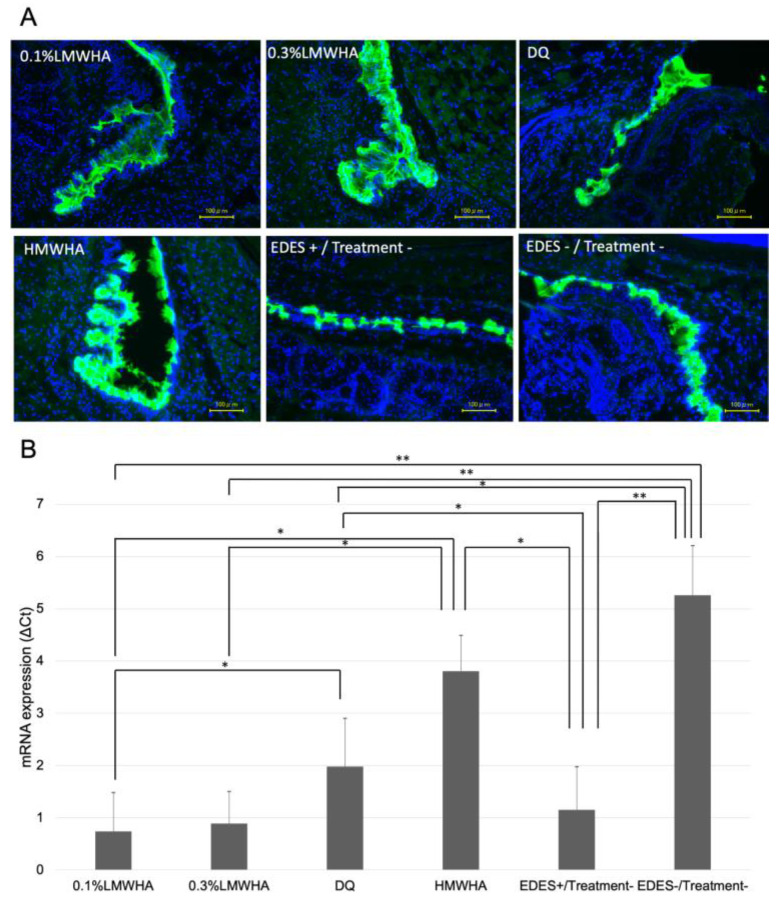
Comparison of Muc5AC immunohistochemistry and mRNA expression in the conjunctiva. (**A**) Representative immunohistochemistry image showed marked decrease of immunostaining in the EDES+/Treatment− group. Fluorescein isothiocyanate (green) and 4’,6-Diamidino-2-phenylindole (DAPI) (blue) were used for Muc5AC and nuclear staining respectively. (**B**) The Muc5AC mRNA expression (ΔCt) in the EDES+/Treatment− group was significantly lower than the HMWHA, DQ, and EDES−/Treatment− groups. In addition, the Muc5AC mRNA expression in the HMWHA group was significantly higher than the 0.1% LMWHA, 0.3% LMWHA, and EDES+/Treatment− groups. * and ** represent *p* < 0.05 and *p* < 0.001, respectively. LMWHA, low molecular weight hyaluronic acid (HA); DQ, diquafosol sodium; HMWHA, high molecular weight HA; EDES, environmental dry eye stress.

On the fourth day after EDES exposure, ocular tissues were collected, and Muc5AC immunostaining and mRNA expression analysis were performed (Figure 6). Immunostaining showed a decrease in staining in the EDES+/Treatment− group compared to the other groups, and stronger staining in the HMWHA group. The mean expression of Muc5AC in the HMWHA group was significantly higher than in the 0.1% LMWHA group (*p* = 0.011), the 0.3% LMWHA group (*p* = 0.014), and the EDES+/Treatment− group (*p* = 0.022). The 0.1% LMWHA group (*p* <0.001), the 0.3% LMWHA group (*p* < 0.001), and the DQ group (*p* < 0.001) showed significantly lower values than the EDES−/Treatment− group. The DQ group showed a significantly higher value than the 0.1% LMWHA group (*p* = 0.037).

## 3. Discussion

In this study, we investigated the therapeutic effects of HMWHA eye drops, LMWHA eye drops and DQ eye drops in an EDES-induced dry eye mouse model. One hypothesis of our study was that, due to their pronounced viscoelasticity, HMWHA eye drops would stay longer on the ocular surface and provide improved lubrication, both resulting in a reduced staining score. Another hypothesis was that, due the larger chain length, the HMWHA molecules might have had a higher potential than LMWHA molecules to entangle with and substitute gel forming Muc5AC chains, which might result in improved tear film stability. Moreover, if the HMWHA were to provide a significant anti-inflammatory effect on the ocular surface, this could result in the protection of the lacrimal glands and goblet cells against T cell-mediated damage and be reflected in increased levels of tear volume and Muc5AC.

In the EDES+/Treatment− group, tear secretion decreased, TBUT shortened, and vital staining score increased after EDES exposure. These results indicated that EDES model did cause dry eye.

Tear secretion significantly decreased in the 0.1 and 0.3% LMWHA groups, but not in the DQ and the HMWHA groups. Since diquafosol sodium induces secretion of aqueous fluid from the conjunctiva via the P2Y2 receptors, it is possible that the tear secretion was maintained even after EDES exposure in the DQ group. HMWHA eye drops may have a stronger effect of retaining water on the ocular surface than thee LMWHA eye drops due to the high viscosity.

TBUT decreased in the LMWHA and DQ groups due to EDES exposure but not in the HMWHA group. TBUT in the HMWHA group after EDES exposure was significantly longer than the LMWHA and DQ groups. This suggests that, in the HMWHA group, in addition to maintenance of the tear volume, high molecular weight HA may remain on the ocular surface for a long time and stabilize the tear film for a longer time.

Vital staining scores by fluorescein and lissamine green stainings were performed to evaluate the ocular surface abnormalities. In all treatment groups, the staining score was worsened by EDES exposure. However, the mean fluorescein staining score in the HMWHA group after EDES exposure was significantly lower than the 0.1 and 0.3% LMWHA groups. Similarly, the mean lissamine green staining score in the HMWHA group after EDES exposure was significantly lower than the 0.1 and 0.3% LMWHA and DQ groups. These results suggest that the HMWHA drops protect the corneal epithelium more than other treatment groups by increasing the tear volume and prolonging the BUT. HA is a linear sugar chain existing extracellularly, binds to various molecules on the cell surface, and is involved in various processes of wound healing via signal transmission [20]. A previous study evaluating skin wound healing in diabetic rats has reported that topical administration of HMWHA promoted wound healing over LMWHA [21]. This suggests that HMWHA itself may have been effective in promoting wound healing even in ocular surface abnormalities due to dry eye.

When the corneal subepithelial nerves were observed using confocal microscopy, the EDES +/Treatment− group showed a significant decrease in nerve density compared to all other groups. This is consistent with a previous report and indicates that the EDES model is a promising model for dry eye studies [11]. Contrary to expectations, there were no significant differences between the four eye drop groups. The reason for this discrepancy was believed to be due to large variations between individuals and the duration of exposure to EDES being as short as 3 days. It is necessary to conduct other studies using eye drops for a longer period of time in the future.

In addition to the observation of the subepithelial nerve plexus, the subepithelial density of presumably dendritic cells was also examined. Interestingly, the dendritic cell density in the HMWHA group was significantly lower than that of the EDES+/Treatment− group. On the other hand, the other treatment groups showed no differences compared with the EDES+/Treatment− group. It is known that HMWHA and LMWHA have different effects on inflammation due to differences in molecular weight. LMWHA elicits a pro-inflammatory response, increasing expression of various inflammatory cytokines and protein production [22,23,24]. This response is primarily activated through CD44, but other receptors, such as Toll-like receptors (TLRs), are also involved in hyaluronan signaling [25]. Specifically, LMWHA activates TLR2 and TLR4 to initiate Myeloid differentiation primary response 88 (MYD88)/NFκB signaling and induces the production of proinflammatory cytokines and chemokines, such as IL-6 and TGFβ [25,26,27,28]. On the other hand, HMWHA forms a large polymer, thus exhibiting anti-inflammatory and immunosuppressive properties. It has been reported that TLR4, TLR2, MyD88, and NFκB expression and protein synthesis were significantly reduced in synovial cells of osteoarthritis model mice [29]. It is possible that HMWHA masks TLR2 and TLR4 via its polymerized structure and, subsequently, prevents stimulation of these receptors [30].

**Figure 7:**
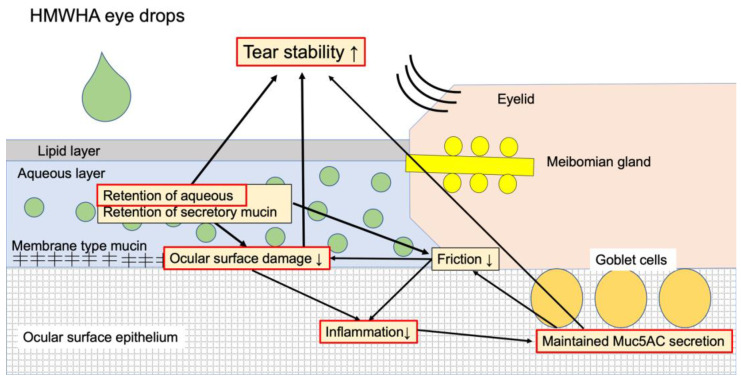
Suggested mechanisms of HMWHA eye drops in the treatment of dry eye disease. HMWHA eye drops have two main functions. One is the retention of aqueous and secretory mucin in the aqueous layer. This effect reduces friction between the eyelids and the ocular surface and, consequently, reduces the ocular surface damage and inflammation. The tear film stability is enhanced by the reduction of ocular surface damage and the retention of aqueous and secretory mucin in the aqueous layer. The second function is the anti-inflammatory effect, which can maintain secretory mucin Muc5AC expression. This action also increases the stability of the tear film. The parts surrounded by red boundaries are those mechanisms proved by the current research, and the other parts are speculative mechanisms which need to be clarified in future studies. HMWHA, high molecular weight hyaluronic acid (HA).

Tear film instability is deeply involved in the core mechanism of dry eye [9,10]. In dry eye disease due to the VDT work, evaporation of tears is increased because of a decrease in the blink frequency, and lacrimal dysfunction causes a decrease in tear secretion [3]. Subsequently, increased friction on the ocular surface and ocular surface damage cause a decreased expression in secretory mucin MUC5AC and membrane mucin, resulting in the tear film instability. It is presumed that HMWHA exerts a therapeutic effect on dry eye due to two factors. One is the anti-inflammatory property due to the above-mentioned molecular biological effect, and the other is water retention property. The water retention property also secondarily suppresses inflammation on the ocular surface by reducing friction between the eyelid and the ocular surface and reducing the ocular surface damage. When HMWHA eye drop treatment is applied to the concept of Tear Film Oriented Therapy as suggested by the Asia Dry Eye Society, HMWHA is thought to improve the mucin layer and improve the epithelial layer. LMWHA enhances IFNγ induction in mouse articular chondrocyte cultures stimulated with lipopolysaccharide, but HMWHA has no effect on it [29]. Previous reports suggest that dry eye increases IFNγ in conjunctival tissues, resulting in decreased goblet cell density and decreased Muc5AC expression [31]. The higher expression of Muc5AC mRNA in the HMWHA group than in the LMWHA group in this study may be due to the IFNγ-mediated action of HMWHA. Figure 7 summarizes the suggested mechanism of HMWHA eye drops in the treatment of dry eye disease. In the future, it will be necessary to elucidate the detailed molecular biological mechanism of HMWHA in dry eye disease.

HA is also closely associated with superoxide dismutase (SOD), which has a role in improving inflammation during wound healing [32]. SOD is also present in mitochondria and is an important antioxidant defense mechanism. Extracellular SOD has a polycation matrix binding domain that binds to HA and partially inhibits inflammation by preventing superoxide-mediated fragmentation of HA. In addition, HA also protects tissues from ROS damage by acting as a scavenger against ROS and free radicals generated by the inflammatory response. Even in EDES model mice, the oxidative stress response in the corneal tissues may be different depending on the eye drops, and further studies are needed in the future.

Many office workers show short BUT-type of dry eyes. It has been reported that the level of MUC5AC in tears of short-BUT office workers is lower than the office workers without dry eyes, and that patients with strong subjective symptoms have lower Mc5AC levels than patients with weak subjective symptoms [4]. In this study, immunostaining and RT-PCR in the EDES+/Treatment− group confirmed a decrease in Muc5AC protein and mRNA expression. Conjunctival tissues in the HMWHA group showed the highest mRNA expression of Muc5AC after EDES exposure among the four eye drop groups, indicating that HMWHA eye drop group was least susceptible to EDES. Although the detailed mechanism is unknown, it was speculated that healthy conjunctival epithelium was maintained by the anti-inflammatory effects of HMWHA and by the promotion of epithelial repair.

This study has several limitations. First of all, eye drop instillation was performed twice a day based on previous studies in canines [33]. As a result, it was shown that HMWHA shows a long lasting effect with a low frequency of eye drops. HMWHA eye drops may be suitable not only for humans but also for the treatment of dry eye in animals in which eye drops cannot be instilled many times a day. However, in clinical use in Japan, HA ophthalmic solution is prescribed 5 to 6 times a day, and diquafosol sodium ophthalmic solution is prescribed 6 times a day as the standard treatment. Therefore, the effects of LMWHA and DQ may be changed by increasing the number of instillations. It is also necessary to perform studies with a higher frequency of instillations in the future. Finally, in this study, in order to avoid the influence of sex hormones, the experiments were performed only with the male mice. Dry eye is often more prevalent in women, and studies with female mice will also be needed in the future.

The standard deviation in each group was relatively high especially in relation to the vital staining scores. The reason for this may be the problem inherent to the evaluation of the vital staining scores in mice in general and in the current environmental dry eye stress model. In our experience, it was confirmed that unpredictable events, such as quarrel between mice and itching behavior after eye drop instillations, increased after stress, which might have led to an increase in standard deviation of the vital staining scores. Further studies are needed to confirm our results with different dry eye models in the future, including the Cu/Zn superoxide dismutase 1 (SOD1) knock out mice which have been shown to be good models of age related dry eye disease [34] or the Non Obese Diabetes (NOD) mice which were shown to be good models for severe forms of dry eye disease, such as Sjogren’s syndrome [35].

In the current study, HMWHA eye drops showed higher therapeutic effect on dry eyes than other eye drops in the EDES dry eye model mice. However, in actual clinical practices, the duration of VDT work may become longer, or the effects of severe air conditioning may be added. In addition, although all eye drops used in the study are preservative-free, it is necessary to examine the tolerability, including the side effects of eye drops in future studies.

In Japan, office workers with dry eyes are instructed to use eye drops before VDT work to improve the ocular surface inflammation and epithelial damage. For this reason, in this experimental protocol, topical treatment using eye drops was performed for 3 days before EDES exposure. Strictly speaking, the present results reflect both the dry eye preventive effect and the therapeutic effect of eye drops. In the future, studies which evaluate only the therapeutic effects will be essential and provide invaluable information.

## 4. Materials and Methods

### 4.1. Animals and Experimental Procedure

**Figure 8:**
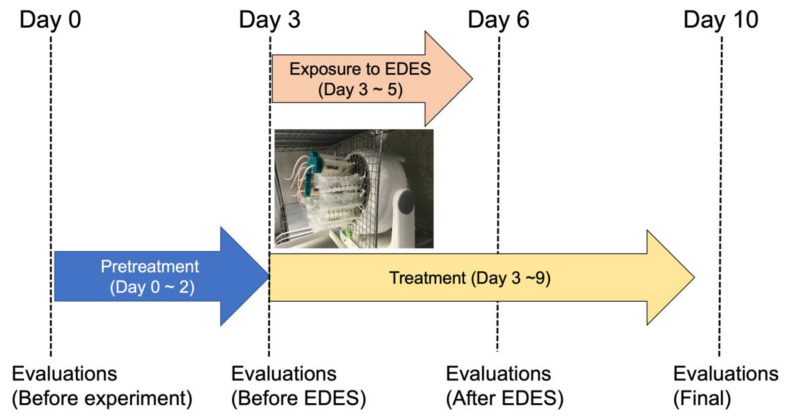
Experiment flow. Eye drops treatment was started 3 days before exposure to EDES (day 0–2) and continued for another 3 days during exposure to EDES (day 3–5). Evaluations of vital staining, tear secretion, and tear breakup time were performed at four time points. On the fifth day after EDES exposure (day 10), in vivo confocal microscopy was performed; after euthanasia, the conjunctival tissue was collected. The photo shows mice exposed to environmental dry eye stress. EDES, environmental dry eye stress.

Sixty 10-week-old C57BL/6 male mice were randomly divided into 6 groups of 10 mice per group. Instillations were started twice a day three days before EDES (day 0–2). Sample size calculations were carried out using the G*Power software (Heinrich Heine, Düsseldorf University, Düsseldorf, Germany) by specifying input parameters including the standard deviation, effect size, and type 1 error [36]. The eyes were similarly instilled for three days during the exposure to EDES (day 3–5) and for four days thereafter (day 6–9); the mice were euthanized, and the tissues were collected on the fifth day after EDES (day 10). The mice were divided into six groups, as follows: (1) 0.1% low molecular weight HA ophthalmic solution (0.1% hyalein mini, Santen, Osaka, Japan); (2) 0.3% low molecular weight HA ophthalmic solution (0.3% hyalein mini, Santen); (3) 3% diquafosol sodium ophthalmic solution (Diquas, Santen); (4) 0.15% high molecular weight HA ophthalmic solution (Comfort Shield, i.com medical, München, Germany); (5) exposure to EDES without treatment; and (6) no exposure to EDES without treatment. Experiment flow was shown in Figure 8.

All studies were performed in accordance with the Association for Research in Vision and Ophthalmology (ARVO) Statement for the Use of Animals in Ophthalmic and Vision Research. The Animal Experimentation Ethics Committee of the Keio University School of Medicine approved the current research procedures (08067).

### 4.2. Application of Environmental Dry Eye Stress

All mice were exposed to EDES for five hours per day (9:00 a.m. to 2:00 p.m.) for three days. The implementation of EDES is described in detail in our previous study [11]. Briefly, the mice were isolated in singular equal-sized small compartments and exposed to a stable, continuous airflow (4 m/s) produced by an 18-cm-diameter electric fan. The fan was set 5 cm away from the mice, mean relative humidity in the room was set to 25 ± 5%, and mean room temperature was fixed at 23 ± 2 °C.

### 4.3. Aqueous Tear Secretion Quantity and Tear Film Stability Assessment

For the tear volume measurement, a cotton thread test was performed based on the previous report [34]. The measurement of tear volume was performed under anesthesia. A cotton thread (Zone-Quick, Showa Yakuhin Kako Co., Ltd., Tokyo, Japan) was inserted into the lateral canthus of the mouse, and the wet length of the cotton thread was measured after 15 s. Then, the value was corrected with the weight of the mouse, and the tear volume was calculated.

TBUT measurement was performed before measurement of the vital staining score. First, under anesthesia, 1 μL of 2% fluorescein staining solution was instilled, and excess solution was removed from lateral canthus. Thereafter, the BUT was measured. The measurement was performed three times, and the average value was adopted.

### 4.4. Ocular Surface Vital Staining Assessment

For evaluation of the fluorescein staining score, a 2µL of 2% fluorescein staining solution was instilled using a micropipette, and the excess liquid was absorbed from the lateral canthus. Thereafter, ocular surface was observed using a portable slit lamp microscope to score staining points. Next, the fluorescein stain was washed away with 5 µL of physiological saline, and 2 µL of 2% lissamine green stain was similarly applied by eye drops and stained. For both the fluorescein staining and the lissamine green staining scores, only the corneal portion was scored from 0 to 5 points using the Oxford scale. Vital staining photographs were recorded in JPEG format using a microscope connected to a digital camera with the same settings maintained for all mice.

### 4.5. In Vivo Laser Scanning Confocal Microscopic Examination and Image Analysis

The observation of the corneal nerves of the mice was performed by using in vivo confocal microscopy (IVCM) and the Heidelberg Retina Tomograph II-Rostock Cornea Module (Heidelberg Engineering GmbH, Dossenheim, Germany) in the same manner as previously reported [11,37]. Prior to testing, mice were anesthetized by intraperitoneal administration of pentobarbital (40 mg/kg). After anesthesia was sufficiently induced, a carbomer 2% gel (Comfort gel, Bosch & Lomb, Berlin, Germany) was placed on the cornea, and the examination was performed so that the cornea was not dried or damaged. High resolution real-time images were obtained by IVCM which consists of 384 × 384 pixels covering an area of 400 × 400 μm (horizontal × vertical) with a lateral resolution of 1 μm/pixel.

Scanning was performed from the corneal epithelial side, and the subepithelial nerve plexus was photographed. The examination time was within 5 min per eye and 6 to 8 areas were photographed. Consisting of 100 images per sequence, about 600–800 confocal images were taken per eye. All sequences were stored as JPEG format images, among which eight non-overlapping representative images were selected per eye. Based on past reports [11,37], two researchers independently measured nerve density and dendritic cell density. For the current study, subbasal nerve fiber density (NFD) and dendritic cell density were evaluated.

(1) NFD was evaluated by measuring the total length of the corneal subbasal nerve fiber in the frame (160,000 µm^2^), as defined in a previous study [18]. After semi-automatic marking of the corneal subbasal nerve in each frame, NFD was automatically measured by the NeuronJ plug-in for ImageJ software (National Institutes of Health, Bethesda, MD, USA). Four different representative images of each cornea were analyzed and subbasal NFD was measured in pixels. The average NFD for each cornea was calculated by averaging these sums. Data were reported as density (µm/mm^2^) ± standard deviation (SD).

(2) The density of presumably dendritic cell was also examined from the pictures in the scanned image. Dendritic cells are sometimes difficult to distinguish from keratocytes in the corneal stroma. Therefore, when counting dendritic cells, we chose the scanned images from the subbasal area and basal epithelial layer at a position shallower than the corneal stroma. If it was difficult to distinguish from the cell morphology whether a cell was a dendritic cell or not, so we judged the cells to be dendritic cells by checking the scan with slightly different focal depths above and below the cell to see the entire cell shape. Presumed dendritic cell density was evaluated using six representative images.

### 4.6. Collection of Cornea and Conjunctival Tissues

The mice were euthanized after the experiments were completed, and the eyes including the eyelids were excised, stored, and frozen in an optimal cutting temperature (OCT, Sakura Finetek, Tokyo, Japan) compound and used for immunostaining. For real-time RT-PCR, conjunctival tissues were trimmed from the eye ball using fine scissors and immediately immersed in RNA later (Applied Biosystems, Carlsbad, CA, USA).

### 4.7. Immunohistochemistry for Conjunctival Muc5AC

Mouse eyeballs stored in OCT compound were sliced to a thickness of 12 µm and mounted on glass slides. Slides were dried at room temperature for 30 min. The samples were fixed with 4% paraformaldehyde for 20 min at room temperature and then washed 3 times with Phosphate-buffered saline (PBS) for 5 min. Blocking was performed with 5% normal donkey serum in PBS for 1 h at room temperature. Thereafter, 100 µL of a primary antibody (anti-Mac5AC mouse monoclonal antibody: ab3649, Abcam, Cambridge, MA, USA) (1:100) was reacted at 4 °C. overnight. Mouse IgG Isotype was used as a control. After the reaction with the primary antibody, the slides were washed three times with PBS. The secondary antibody (FITC-affinity pure donkey anti-mouse IgG: 715-695-150, Jackson Laboratory, Bar Harbor, ME, USA) (1:200) was reacted at room temperature for 1 h. After washing with PBS, nuclear staining with 4’,6-diamidino-2-phenylindole (DAPI) (19178-91, Nacalai Tesque, Kyoto, Japan) (1:2000) was performed at room temperature for 5 min. After washing with PBS, the samples were sealed and observed with a microscope.

### 4.8. Quantitative Real-Time PCR for Conjunctival Muc5AC

Mouse conjunctival tissues were preserved overnight at freezer (−80 °C) in RNAlater (Applied Biosystems, Waltham, MA, USA) after prompt excision. Tissues were then transferred into Isogen reagent (Nippon Gene Co., Tokyo, Japan) and homogenized well. Total RNA was extracted, cleaned up, and treated with DNase using the RNeasy mini kit (Qiagen Inc., Valencia, CA, USA). cDNA synthesis was performed using the ReverTra Ace qPCR RT kit (TOYOBO, Tokyo, Japan). SYBR Greene based quantitative real-time PCR was performed using the Step-OnePlus system (Applied Biosystems, Waltham, MA, USA) with THUNDERBIRD SYBR qPCR Mix (TOYOBO, Tokyo, Japan). Mouse glyceraldehyde-3-phosphate dehydrogenase (sense: 5’-TGACGTGCCGCCTGGAGAAA-3’, antisense: 5’-GACTTCCCGTAGAACCCGATGTGA-3’), Muc5ac (sense: 5’-AAAGACACCAGTAGTCACTCAGCAA-3’, antisense: 5’-ACCAAACTGTGACTGAAGGGTC-3’) primers were used as template. Data were normalized to glyceraldehyde-3-phosphate dehydrogenase.

### 4.9. Statistical Analysis

To assess the effects of time-course changes in each group, a one-way repeated measures ANOVA was performed to analyze tear quantity, vital staining scores, and corneal subbasal nerve parameters at different time points in each group. To compare the values at the same time-points between different groups, one-way ANOVA was applied. Tukey’s test was then performed as a multiple comparisons test (post-hoc test). A *p*-value less than 5% was considered to be a statistically significant.

## 5. Conclusions

The HMWHA ophthalmic solution had a higher therapeutic effect in EDES induced dry eye model mice than the LMWHA and DQ ophthalmic solution, possibly due to the anti-inflammatory effects and maintenance of the secreted mucin Muc5AC.
